# Validating Automatic Concept-Based Explanations for AI-Based Digital Histopathology

**DOI:** 10.3390/s22145346

**Published:** 2022-07-18

**Authors:** Daniel Sauter, Georg Lodde, Felix Nensa, Dirk Schadendorf, Elisabeth Livingstone, Markus Kukuk

**Affiliations:** 1 Department of Computer Science, Fachhochschule Dortmund, 44227 Dortmund, Germany; markus.kukuk@fh-dortmund.de; 2 Department of Dermatology, University Hospital Essen, 45147 Essen, Germany; georg.lodde@uk-essen.de (G.L.); dirk.schadendorf@uk-essen.de (D.S.); elisabeth.livingstone@uk-essen.de (E.L.); 3 Institute for AI in Medicine (IKIM), University Hospital Essen, 45131 Essen, Germany; felix.nensa@uk-essen.de; 4 Institute of Diagnostic and Interventional Radiology and Neuroradiology, University Hospital Essen, 45147 Essen, Germany

**Keywords:** explainable AI, bias discovery, concept attribution, saliency map, computational histopathology, malignant melanoma, basal cell carcinoma, squamous cell carcinoma, intra-epidermal carcinoma

## Abstract

Digital histopathology poses several challenges such as label noise, class imbalance, limited availability of labelled data, and several latent biases to deep learning, negatively influencing transparency, reproducibility, and classification performance. In particular, biases are well known to cause poor generalization. Proposed tools from explainable artificial intelligence (XAI), bias detection, and bias discovery suffer from technical challenges, complexity, unintuitive usage, inherent biases, or a semantic gap. A promising XAI method, not studied in the context of digital histopathology is automated concept-based explanation (ACE). It automatically extracts visual concepts from image data. Our objective is to evaluate ACE’s technical validity following design science principals and to compare it to Guided Gradient-weighted Class Activation Mapping (Grad-CAM), a conventional pixel-wise explanation method. To that extent, we created and studied five convolutional neural networks (CNNs) in four different skin cancer settings. Our results demonstrate that ACE is a valid tool for gaining insights into the decision process of histopathological CNNs that can go beyond explanations from the control method. ACE validly visualized a class sampling ratio bias, measurement bias, sampling bias, and class-correlated bias. Furthermore, the complementary use with Guided Grad-CAM offers several benefits. Finally, we propose practical solutions for several technical challenges. In contradiction to results from the literature, we noticed lower intuitiveness in some dermatopathology scenarios as compared to concept-based explanations on real-world images.

## 1. Introduction

Goodman et al. [1] described reproducibility of results (or replicability) as “the production of corroborating results in a new study, having followed the same experimental methods”. It is a major principal of the scientific method, as it is used to filter chance findings and unscientific claims [2]. Therefore, studying and understanding factors limiting the reproducibility of findings is an important and well-investigated topic in scientific research [3,4]. In particular, for the medical field, recent studies [5,6] raise concerns regarding reproducibility and transparency for medical artificial intelligence (AI), showing that deep learning (DL) models applied to histopathology can learn unwanted and spurious correlations with hidden variables such as clinical or meta data. This can be problematic if they are not causally related to the actual classification task.

Explainable Artificial Intelligence (XAI) seeks to resolve or at least mitigate those issues [7] and therefore plays an important role, not only in DL in general, but also in the medical field. For example, for computational histopathology, recent reviews have emphasized the importance of XAI for the translation to clinical practice [8,9], where patient safety is paramount and medical practitioners are confronted with judicial implications [10] when AI methods fail to fulfill legal or regulatory requirements. Hägele et al. [7] have investigated the role of a pixel-wise visual explanation method for the field of histopathology and identified the following factors limiting classification accuracy: label noise, class imbalance, limited availability of labelled data, and several latent biases. Especially biases, such as measurement bias (e.g., from stain variance), class sampling ratio bias, sampling bias and class-correlated bias often pose theoretical and practical limits to the quality of a model’s predictions. If left untreated, they are a major obstacle as they reduce generalization [11]. However, methods that explain AI results might address this by giving insights into a model’s decision process.

Several authors have developed solutions specifically for bias detection and discovery in computer vision. Here, we use the term bias detection for the detection of a predefined bias type, while bias discovery refers to the detection of previously undefined biases. Some methods rely on bias vectors in a latent space. Li and Xu [12] have combined generative adversarial networks (GANs) with orthogonalization penalty. Alternatively, informative regions can be deliberately manipulated using GANs [13,14,15] and other generators [16,17]. Del Tejo Catalá et al. [18], Zhang et al. [19], and Bissoto et al. [20] have studied biases by masking informative regions. Some methods make use of clustering. Four extensions [21,22,23,24] of the spectral relevance analysis (SpRAy) framework [25] cluster layer-wise relevance propagation (LRP) heatmaps (synonymous: saliency maps, pixel attribution). The unsupervised discovery of bias in deep visual recognition models (UDIS) algorithm by Krishnakumar et al. [26] clusters feature embeddings. Reimers et al. [27,28] have verified bias concepts using structural causal model and Reichenbach’s common cause principle. Two methods, the bias discovery score based on conditional probabilities by Tian et al. [29], and the activation ratio threshold of InsideBias [30], rely on neuron activations. REvealing VIsual biaSEs (REVISE) detects biases based on attribute distributions [31]. As we will show for the field of histopathology, however, all methods for bias detection and discovery suffer from incompatibility, limited utility, additional complexity, or require prior knowledge (Section 4.3).

In a broader context, XAI addresses challenges such as explanation, interpretability, meaningfulness, accuracy, and knowledge limits [32]. Most of the research on XAI in DL is focused on pixel-wise methods such as LRP [33], Local Interpretable Model-agnostic Explanations (LIME) [34], or Gradient-weighted Class Activation Mapping (Grad-CAM) [35] that display their results in form of a heatmap. If a model is biased, XAI should somehow indicate noticeable problems regarding its decision criteria. XAI methods are therefore basically an alternative to bias discovery methods. However, they are not primarily designed for this specific task. Therefore, the question arises as to whether and how biases are discovered by a specific XAI tool and how well the tool fits the user needs. Some evaluations can be found for MMD-critic [36], Grad-CAM [36,37], Kernel SHapley Additive exPlanation (SHAP) [38], testing with concept activation vectors (TCAV) [37], LRP [7], and further saliency methods [39]. Korbar et al. [40] have applied (Guided) Grad-CAM under the broader context of visualization and insight. There is a controversial debate about the coherence of established saliency maps [41,42,43], which has resulted in algorithmic improvements [44,45]. Thus, histopathologists face controversial advice from research when considering saliency maps for a specific task. Another criticism is that they are “far from the semantics of the physicians, who focus on affected region size, shape and aspect” [46]. In case of explanations based on heatmaps, the histopathologist must look at all heatmaps to interpret highlighted pixel areas. Interpretation and logical combination of those areas is then needed to develop an abstract understanding in terms of histopathological concepts. In other words, histopathologists are required to build an abstraction on a semantic level. Tong and Kagal [37] have already argued that this might be a potential pitfall. Furthermore, the approach appears laborious, time-consuming and tiring [23].

Recent work in XAI has proposed concept-based approaches [47]. TCAV explains DL models using human-friendly notions [48]. Several authors have tried to achieve further algorithmic advancements over the work of Kim et al. [48]. On the one hand, modifications of the TCAV algorithm can be found [49,50]. Some authors have gone even further and have extended concepts to hierarchies [51], structural [52] or causal [53] graphs, or functional relationships [54]. On the other hand, several authors have improved upon TCAV by automating the concept discovery process [55,56,57,58,59]. Concept-based explanation has been applied to different medical domains, including dermatoscopy based skin cancer classification [60], infectious keratitis [61], cardiac magnetic resonance imaging [62], retinopathy of prematurity [63], glaucoma in optical coherence tomography [64], cardiac MRI [65], or electronic health records [66]. Applications to histopathology can be found in the evaluation of the TCAV [67], Regression Concept Vectors (RCV) [46], and Similar Medical Images Like Yours (SMILY) methods [68,69]. However, such methods rely on user-predefined concepts, which may be challenging to establish in the first place and additionally may in themselves contain human biases [23,37,55].

All bias discovery and XAI methods considered so far do not meet the needs of digital histopathology [7]. However, to our knowledge automated concept-based explanation (ACE) proposed by Ghorbani et al. [55], has not been studied for its relevance for the histopathological field. ACE is a promising method capable of helping with explaining and interpreting AI results to researchers as well as practitioners. ACE automatically extracts visual concepts from data [55]. Since concepts are algorithmically created, human confirmation bias is reduced [55]. Furthermore, what is displayed appears to be in line with what the histopathologist has in mind from a medical/biological perspective, as the result is shown “in terms of human-understandable, usually higher-level, ideas” [37]. The interpretation process requires less effort (also see [68]) and less logical arguments and therefore appears more intuitive [70]. Graziani et al. [46] have argued that this semantic match and intuitive interaction make concept-based explanation methods an important complementary technique to heatmap-based methods in histopathology. The authors have also reasoned that it can lead to higher trust in the histopathological context [46,68].

Therefore, our research question is: To what extent is ACE alone, or in complement with a heatmap based method technically valid to address a common challenge, namely bias discovery, in computational histopathology? Our work builds upon the work by Hägele et al. [7] and extends their research approach by strictly following the design science methodology [71,72]. Our work contributes to deeper insight in the following ways:

- We apply two XAI methods to histopathology: ACE and Guided Grad-CAM as examples for explanations on model level and image level, respectively.
- We demonstrate the validity of ACE for bias discovery in the histopathological context (malignancy detection and cancer subtyping). ACE offers histopathologists an additional diagnostic tool for helping with explaining and interpreting the results of convolutional neural networks (CNNs).
- We balance advantages and disadvantages for using either method for discovering biases in AI systems based on DL.
- We observe that for dermatopathology, explanations obtained from ACE lack the high degree of intuitiveness often found for real-world (ImageNet) applications.
- We offer technical insights into the application of ACE on histopathology.

## 2. Materials and Methods

The section elaborates on our research methodology and the XAI methods studied, as well as on our experimental design and setup.

### 2.1. Design Science Research Methodology

We adopt the definition of Hevner and Chatterjee [73], according to which an information technology (IT) artifact is a construct, model, method, instantiation, or design theory that was artificially created by humans to solve a specific problem. Our research question focuses on the evaluation of such an IT artifact (ACE) in the histopathological context. In information systems research, the design science research (DSR) approach by Hevner et al. [71] has become widely established as an appropriate research methodology and is therefore our methodology of choice.

More specifically, we evaluate prototypes using the demonstration pattern. The pattern verifies the validity of the solution in a predefined set of situations [74]. Those situations must be justified, and the evaluated artefact components must be specified. As the artifact might work in other situations as well, this is seen as a first (weaker) form of evaluation. How well an artefact works is not quantified in the first step. However, as a second step, more rigorous (e.g., quantitative) evaluations should thus follow [75].

The quality we examine is validity, which according to Gregor and Hevner [72] means that the artifact “works and does what it is meant to do; that it is dependable in operational terms in achieving its goals” [72]. Applied to our investigation, this means that to be technically valid, ACE explanations must indicate if the model is biased. In the same sense, they must show the absence of biases in case of unbiased models to increase confidence in the decision criteria.

### 2.2. Explainable AI Methods

This section outlines the two XAI methods applied in this study. While ACE [55] represents a method from the category of global explanations on model level, Guided Grad-CAM [35] represents local explanations on image level using a pixel-wise heatmap visualization.

#### 2.2.1. Automated Concept-Based Explanations

In the context of model explanation, Kim et al. [48] understand concepts as “high-level concepts [visual features] that humans easily understand”. For example, one might look for dimples, grass, and sky, when searching for golf balls [55]. Concepts in the histopathological context may include notions such as the count of cavities, nuclei area, nuclei texture, mitotic count, nuclei density, and staining characteristics [46]. While these two examples based on real-world and histopathological images may exhibit a high degree of intuitiveness, there is obviously no guarantee that automatically extracted concepts are likewise intuitive (nor meaningful and coherent [55]).

In general, ACE consists of three steps [55]: first, potential concept patches are automatically extracted by segmenting the original image tile using simple linear iterative clustering (SLIC). SLIC extracts perceptually meaningful atomic regions by iteratively moving cluster centers and reassigning nearby pixels, comparable to k-means clustering. One key feature, a spatially restricted search space, distinguishes it from other alternatives in terms of computational efficiency [76]. In order to acquire concepts of different kinds of complexity, three levels of segmentation are used as proposed in [55]. Second, the intermediate activations as a representation of perceptual similarity are used as the clustering feature. Concept patches are clustered into meaningful concept clusters using k-means clustering. For each concept, only the n segments of smallest $l_{2}$ distance from the cluster center are considered and denoted concept patches [55]. Consequently, concept coherency (perceptual similarity) is increased since in latent space, minimizing $l_{2}$ distance is equivalent to maximizing similarity. In contrast, increasing n would decrease concept coherency. In order to filter out meaningless clusters, Ghorbani et al. [55] have proposed filtering rules. Third, TCAV scores are calculated.

The third step of ACE, testing with TCAV, in turn is carried out in five steps [48]. Let k be a class label, $X_{k}$ all inputs with this label, l a neural activation layer, C a concept of interest, and $S_{C,k,l}\left(x\right)$ the directional derivative. First, a CNN up to layer l is used to transform concept patches into activations. Second, a linear classifier (SVM) is trained on those activations to distinguish between the concept and random counterexamples. Third, the dot product of the vector $v_{C}^{l}$ orthogonal to the decision boundary and the output gradient $\nabla h_{l,k}$ that maximizes prediction of class k is calculated to quantify the sensitivity $S_{C,k,l}\left(x\right)$ to each concept. Fourth, a score quantifies the class-specific importance of a concept. Here, the final quantitative explanation $TCAV_{Q_{C,k,l}}\in \left[0,\;1\right]$ is the fraction of k-class inputs where C has a positive influence on the l-layer activation vector [48]:

(1) $$TCAV_{Q_{C,k,l}}=\frac{\left|\left\{x\in X_{k}:S_{C,k,l}\left(x\right)>0\right\}\right|}{\left|X_{k}\right|}$$

Fifth, a two-sided *t*-test on $TCAV_{Q}$ scores of the concept and random counterexamples from multiple runs is calculated to filter meaningless results. Here, the null hypothesis $H_{0}$ is that $TCAV_{Q}$ scores for the concept patches and for the random counterexamples are equal. A concept is considered meaningful if $H_{0}$ can be rejected with a significance level of α=0.01 [48].

**Figure 1:**
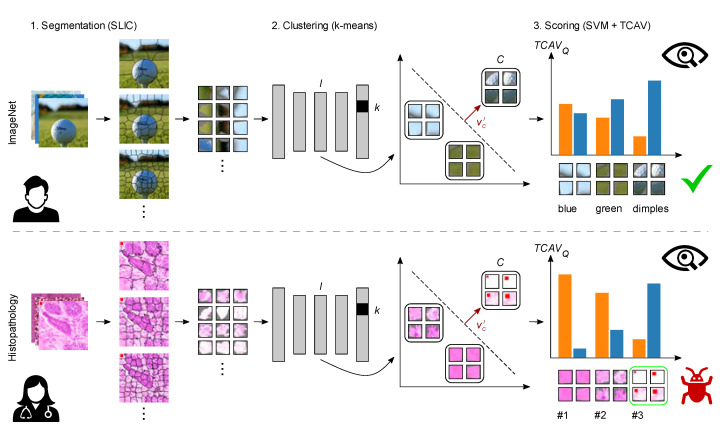
Function principle of ACE applied to real-world (top) and histopathological images (bottom), respectively. First, potential concept patches are automatically extracted from the original images using SLIC clustering. Second, k-means clusters those patches into meaningful concept clusters based on the intermediate activations as a representation of perceptual similarity. Third, a concept activation vector $v_{C}^{l}$ is found using an SVM, and concepts are then ranked by a $TCAV_{Q}$ score, which indicates a concept’s importance for the CNN’s prediction. Other concept quality criteria such as meaningfulness and coherency [55] are not reflected by the score. Extracted concepts and scores are visually inspected by a domain expert. For the binary classification of golf balls, concepts such as dimples, green, and blue are most important, which increases confidence in the model. For classifying cancerous versus non-cancerous tissue ACE helps revealing a planted class-correlated bias, since all members of the most important concept (green frame) share the same feature (red square), unrelated to an expert’s decision. In general, a high $TCAV_{Q}$ score alone does not automatically indicate a bias. For more details on this bias, see Section 2.3.2. Illustration only, based on Ghorbani et al. [55] and Kim et al. [48].

Figure 1 compares ACE applied to real-world and histopathological images. For classifying golf balls, ACE reveals that the model’s decision is largely based on three plausible concepts, thereby increasing confidence in the model. For classifying cancerous versus non-cancerous tissue in derma-histopathological images, ACE helps discovering a class-correlated bias by revealing that the model’s decision is largely based on features unrelated to the task.

For our experiments, we used SLIC-zero [76] for segmentation and set the number of segments to 15, 50, and 80 per image. For clustering, the k-means algorithm was used. The optimal number of clusters was determined using trial and error (see Section 3.3). In accordance with Ghorbani et al. [55], the maximum number n of patches per concept was set to 40. For the calculation of statistical significance and standard deviation, 100 runs were executed. In Section 3, mean scores as well as standard deviations are given. $TCAV_{Q}$ scores were calculated for all possible combinations of ACE concepts and prediction classes. Similar to Kim et al. [48], we visualized those scores using bar charts. Then, these diagrams were visually interpreted, and it was determined on which class a concept had a positive influence.

#### 2.2.2. Pixel-Wise Heatmap: Guided Grad-CAM

Hägele et al. [7] used LRP for fine-grained, pixel-wise explanations. As the authors state that their findings should hold true for any kind of fine-grained heatmap methods, we apply another method from this category, Guided Grad-CAM [35], which was mentioned but not included in their comparison of fine-grained visual explanation methods. (Guided) Grad-CAM is generally more popular in terms of annual citations [77], which makes it reasonable for us to take it into consideration. There is a debate about the sanity of such saliency maps [44,45], which might be irritating for the user. However, Yona and Greenfeld [41] showed that the sanity checks themselves seem to be faulty, which is why we include Guided Grad-CAM in our investigation. Similar to as in [40], the Guided Grad-CAM technique [35] was used to generate pixel-wise heatmaps. The technique combines both, localization of important class-discriminative region, and pixel-precise resolution. For the aim of our study, it should thus perform comparatively to LRP.

(Guided) Grad-CAM calculates the gradient of a class-specific score $y^{C}$ for a set of feature maps $A^{k}$. Let Z be the total number of elements of one feature map. Gradients of a feature map k are aggregated using global average pooling [35]:

(2) $$\alpha_{k}^{c}=\frac{1}{Z}\overset{u}{\underset{i}{\sum }}\overset{v}{\underset{j}{\sum }}\frac{\partial y^{c}}{\partial A_{ij}^{k}}$$

Here, the weight $\alpha_{k}^{c}$ reflects the importance of the feature map k for the prediction of class c. Features with positive influence $L_{Grad-CAM}^{c}$ are filtered using a weighted sum and the Rectified Linear Unit (ReLU) function [35]:

(3) $$L_{Grad-CAM}^{c}=ReLU\left(\underset{k}{\sum }\alpha_{k}^{c}A^{k}\right)$$

This first type of visualization is referred to as Grad-CAM. It is usually superimposed on the image in the form of a colormap. As this visualization map is based on $A^{k}$, it is low-resolution [35]. Thus, a pointwise multiplication with guided backpropagation [78] further adds fine details. This second type of visualization is referred to as Guided Grad-CAM. It takes the form of a grey image with colorful, pixel-precise structures [35].

### 2.3. Experimental Design

We evaluate the validity of ACE for the digital histopathology using instantiation in a set of predefined situations (see Table 1) [74]. Our experimental design is based on Hägele et al. [7]. Despite their synthetic origin, our biases are close enough or equivalent to real-world biases to draw valid conclusions. To ensure that the results are not caused by confounding variables, we only modified the independent variable “bias/no bias”. Other variables were held constant, and results were compared subsequently. In our study, the experimental conditions are not exactly identical to those of Hägele et al. [7]. To be able to better discuss the meaning of our results regarding the preliminary work, we also replicated the experiments on validity of pixel-wise heatmaps.

#### 2.3.1. Feature Visualization

Hägele et al. [7] have analyzed feature visualization on histopathology images in general and have included a quantitative evaluation using the receiver operating characteristic (ROC). While this is appropriate for explanations based on image level, such as LRP, this methodology is not suited for model level explanations provided by ACE. Therefore, we provide a qualitative evaluation of a classifier that we trained to distinguish between melanoma and non-melanoma tissue.

#### 2.3.2. Biases

During the development of an ML model, the AI pipeline can lead to the introduction of a wide variety of unwanted and harmful biases [81]. Hägele et al. [7] have already identified several specific biases that are relevant in histopathology. While we followed their experimental design, we also adopted the more modern bias taxonomy by Srinivasan and Chander [81]. Thus, we consider a skewed class sampling ratio to be a specific type of bias. In this study, the following five biases were examined:

*Class sampling ratio bias:* Hägele et al. [7] have analyzed the impact of different sampling ratios on heatmap visualizations as the generalizability of the model might be impacted by this parameter. Here, we classified cancerous and non-cancerous tissue from squamous cell carcinoma (SCC) whole slide images (WSI). Initially, both tissue types were about equally frequent. To artificially introduce a class sampling ratio bias, we then reduced the probability of a tumor tissue sample by 50%, resulting in a sampling ratio of approximately 2:1. The training data thus represents a distorted tissue distribution.

*Dataset bias:* We considered the suitability of ACE for the discovery of dataset biases. Hägele et al. [7] have defined dataset biases as “biases that affect the entire dataset, i.e., biases that are spread across all classes, including test data”. For their experiments, the authors chose a bias that is location-dependent. They determined the label based on the tissue shown in the patch center. However, ACE is designed to visualize the extracted patches in a cropped manner. There is no location context available, as the location is not visible to the user. ACE is thus not able to show that a bias is specifically limited to the patch center. While it is theoretically possible to visualize the patches without cropping, presentation must be modified specifically for the discovery of this kind of bias. We therefore think that heatmap-based methods are better suited for this task. Consequently, we excluded this experiment from our work.

*Measurement bias:* Instead of the dataset bias on the patch center, we followed a distant approach that affects the whole dataset. In general, measurement bias “is introduced by errors in human measurement, or because of certain intrinsic habits of people in capturing data” [81]. Schmitt et al. [6] have shown that CNNs can unintentionally learn slide preparation and scanner characteristics. In the context of training a CNN to distinguish between melanoma and non-melanoma tissue, we artificially introduced a measurement bias by combining images from two different datasets with different image characteristics into one training dataset. In contrast to the experiment on feature visualization where visual differences between the datasets were eliminated during image preprocessing, we did not use any kind of preprocessing such as stain normalization or color augmentation for image adjustment. The network should thus be able to achieve an accuracy of 100% only based on the dataset characteristics. For example, it could focus on differences in sectioning, fixation, staining, and mounting procedures [6]. Although the measurement bias is closely related to a class-correlated bias, we still include its investigation since the focus lies more on characteristics of the WSI.

*Sampling bias:* We evaluated an artificially introduced sampling bias. This bias generally “arises in a dataset that is created by selecting particular types of instances more than others” [81]. Hägele et al. [7] have accomplished this by classifying cancerous and non-cancerous tissue. However, they have excluded necrotic tissue. We took a similar approach for our study. We classified cancerous and non-cancerous tissue from basal cell carcinoma (BCC) WSIs. However, we intentionally introduced a sampling bias by exemplarily excluding slides with reticular dermis tissue during training. The remaining training data thus mainly consisted of cancerous and inflammatory tissue. For an unbiased model, cancerous cells should be used as a detection criterion, while non-cancerous tissue should be characterized by the absence of cancer-related attributes. In a biased model, however, cancer cells can still be used as a detection criterion for cancerous tissue, but the deviation of distribution for non-cancerous tissue is reflected in the decision criteria in a way that does not match medical diagnostic criteria. Here, the reduction of reticular dermis tissue is a synthetic bias and not medically related to the prediction task. From a technical point of view, tissue type is not of further importance and other tissue types could be chosen instead.

*Class-correlated bias:* Further, a class-correlated bias was evaluated. This form is characterized by “image features that are unintentionally correlated with labels in the dataset” [7]. Hägele et al. [7] have trained a classifier to distinguish between cancerous and non-cancerous tissue. They have introduced the bias by replacing a square region in the upper left corner with a single color on every cancer image patch. We took an equivalent approach for our study. We trained a classifier to distinguish between cancerous and non-cancerous tissue from intra-epidermal carcinoma (IEC, also known as carcinoma in situ). For tumor tiles, a small red square was drawn in the upper left corner of the tile. In this way, we artificially created a class-correlated bias. In a clinical environment, class-correlated biases may arise from contaminations of the microscope glass slides or from preparation artifacts, e.g., if the slides from different classes predominantly come from different institutes. If the preparation or scanning process is slightly different, some images might show visual features which are not present in those of other institutes.

### 2.4. Experimental Setup

#### 2.4.1. Datasets

Two datasets were used in our experiment. The first one, the Histopathology Non-Melanoma Skin Cancer Segmentation Dataset (here referred to as “Queensland”), was acquired in Australia in 2017 and 2018 [80]. It contains 100 shave, 58 punch, and 132 excision biopsies from 67% male and 33% female patients aged between 34 and 96 years (median: 70 years). The slides show hematoxylin and eosin (H&E) stains of 140 BCCs, 60 SCCs, and 90 IECs (three forms of non-melanoma skin cancer). For digitalization, a DP27 Olympus microscope camera at 10× magnification was used [82]. In addition, detailed segmentation maps with information on the tissue types are given. Segmentation includes the classes glands, inflammation, hair follicles, hypodermis, reticular dermis, papillary dermis, epidermis, keratin, background, BCC, SCC, and IEC. The annotation was performed by a dermatopathology laboratory scientist in consultation with a pathologist [82].

The second dataset called The Cancer Genome Atlas (TCGA) Skin Cutaneous Melanoma (SKCM) contains 470 H&E slides of malignant melanoma [79]. Biospecimens were contributed from different tissue source sites worldwide, including from USA, Germany, Poland, Australia, Canada, and Italy [83]. The data comes from 64% male and 38% female patients aged between 15 and 89 years (median: 58 years). Age is unknown for eight patients. Slides were digitized at 40× magnification. For our study, we further digitally resized them to 10× magnification so both datasets have the same scale. There was no segmentation label data available for this dataset. The examination of WSI data was performed by a consensus panel of pathologists [83].

#### 2.4.2. Data Preprocessing

Data was split into training (66.67%), validation (16.67%), and test (16.67%) splits. To train a CNN to distinguish between cancerous and non-cancerous tissue, small tiles of 256 × 256 pixels were randomly extracted from the WSIs during training. To exclude irrelevant background, white areas on each tile was limited to 10% at the maximum. For testing, patches were extracted from the image center to assure reproducibility. For the public dataset that we used, information about the tissue type was available as segmentation maps. Areas of homogeneous kinds of tissue type were labeled by a dermatopathology laboratory scientist in consultation with a pathologist [82]. Using the segmentation map, the binary tile label was determined. If the section contained any cancerous pixels, it was labeled as cancerous. Otherwise, it was considered as non-cancerous. For the experiments on melanoma vs. non-melanoma tissue, segmentation data was missing. Here, a tissue section was manually preselected. During training, small tiles of 256 × 256 pixels were randomly extracted from the section. For testing, patches from the image center were used to assure reproducibility.

Data for the feature visualization experiment came from two different scanners, respectively, two different staining settings. We had to eliminate differences in image characteristics as they would have acted as a confounding variable [6]. We used the color normalization algorithm of Macenko et al. [84]. As color augmentation is always useful for generalization [85], we also applied random HSV color augmentation to the patches during training. Further, training data for all experiments was augmented using random horizontal flip, and rotation (360° maximum) [86]. To avoid technical issues related to the ACE algorithm (see Section 3.3), we also used random cutout with greyscale value 127 [87]. RGB values were then normalized to [0, 1]. The order of image patches in each mini-batch was randomly shuffled during training.

#### 2.4.3. Convolutional Neural Network

**Figure A1:**
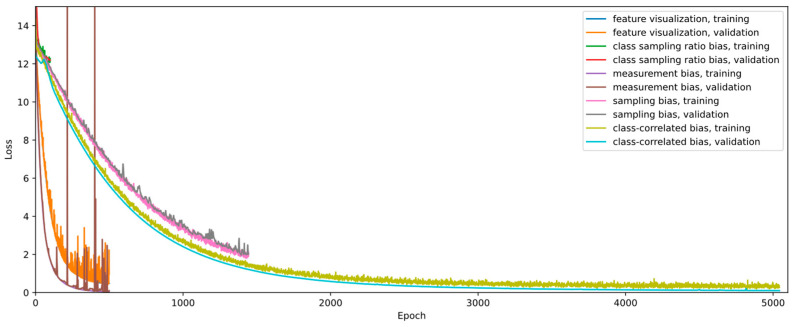
Training and validation loss for all five experimental settings. There is no considerable difference between training and validation loss. Thus, there is no evidence for overfitting on the training split. The generalization on other datasets is out of scope and was not considered. Experiments on class sampling ratio bias were limited to 100 epochs. Validation curves for feature visualization and measurement bias experiments largely obscure the respective training curve.

Hägele et al. [7] used an ImageNet-pretrained CNN called GoogLeNet. While common in medical research, Li and Plataniotis [88] showed that the superiority of ImageNet-based pretraining is not clear. Instead, there is evidence that domain-specific pretraining is more advisable [89]. Since this uncertainty is out of the scope of our research, we deviated from the experimental setup in [7]. Instead of transfer learning, we used “training from scratch”, combined with image augmentation, and L2 regularization. This was enough to avoid potential overfitting on the training split (see Figure A1).

For our experimental setup, we used a simple CNN architecture (see Table 2). Residual connections have shown to improve CNN optimization [90]. We therefore also used residual connections. Our network is very similar to ResNet [90]. However, as the number of classes is dramatically lower (2 vs. 1000), less complexity is needed to describe the detection algorithm. For this reason, we chose a smaller network to avoid unnecessary overfitting. As ReLU is known to suffer from dead neurons due to a gradient of zero [91], we decided to replace it. We used the Swish activation function instead, as it can potentially outperform other alternatives [92].

Model training using the backpropagation algorithm was carried out on a server with two AMD EPYC 7402 24-core processors, one terabyte of random-access memory, an NVIDIA RTX A6000 graphics card, and an Ubuntu 20.04.4 LTS operating system. The model was implemented and trained using Python 3.9.6 (Python Software Foundation, Wilmington, DE, USA) and TensorFlow 2.6.0 (Google LLC, Mountain View, CA, USA) [93]. We optimized the model with Adam using a learning rate of 2 × 10^−4^, and a batch size of 32. Model checkpoint and early stopping with a patience of 50 on validation loss were used. To account for class imbalance, the parameter “class_weight” of the Keras method fit() was used [94]. There is one exception: training with a high number of epochs made our models learn class differences even in a highly class-imbalanced setting. Similar to Hägele et al. [7], we thus limited the number of training epochs to 100 and dropped early stopping and the class weights for the class sampling ratio bias experiment. As reduced classification performance (e. g. due to non-convergence) is to be expected for such a biased model, those modifications were not harmful (and necessary) for our purpose.

## 3. Results

In the following, the results with respect to feature visualization and bias detection/discovery are presented. An overview of all results is given by Table 3.

### 3.1. Feature Visualization

**Figure A2:**
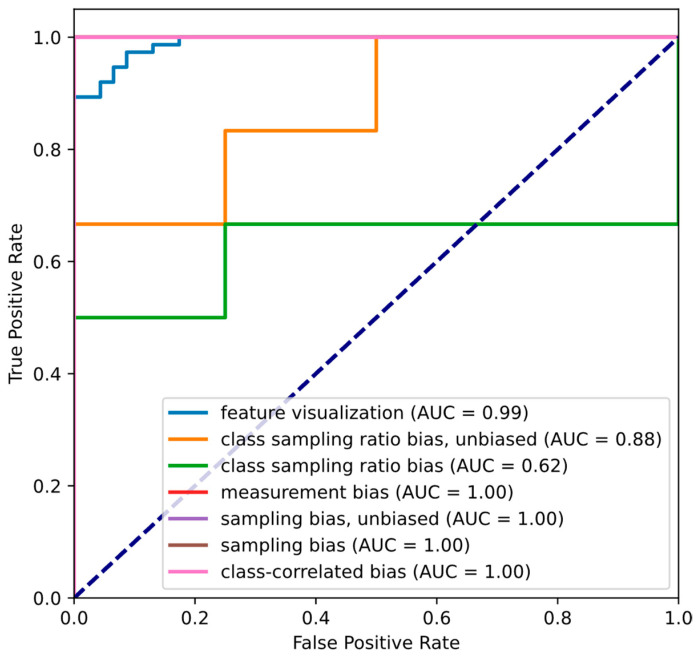
ROC-Curves and ROC AUC scores of the CNNs for all five experimental settings.

Quantitative performance evaluation of the model for feature visualization shows a high classification performance. On the test split, it detected malignant tissue with a balanced accuracy of 94.3%, an area under the curve (AUC) of 0.991, an F1 score of 96.1%, a precision of 94.8%, and a recall of 97.3%. For the ROC curve, see Figure A2.

**Figure 2:**
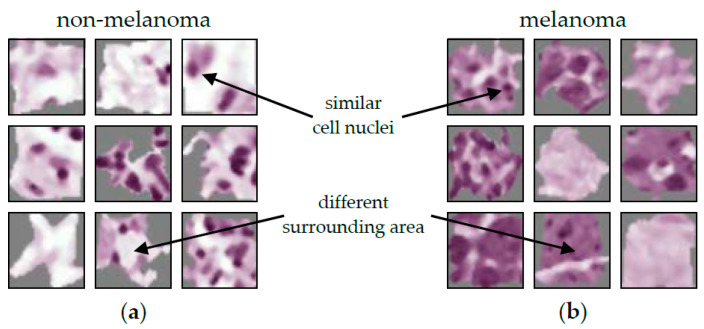
Typical concept patches for the differentiation between malignant melanoma and non-melanoma tumors (BCC, SCC, and IEC). For the entire set of concept patches, see Figure A3. (**a**) Concept for non-melanoma tumors. (**b**) Concept for melanoma tumors. Both concepts seem to share roughly the same cell size, shape, and color. The most obvious criterium to distinguish between the two concepts appears to be the area surrounding the depicted cells.

**Figure A3:**
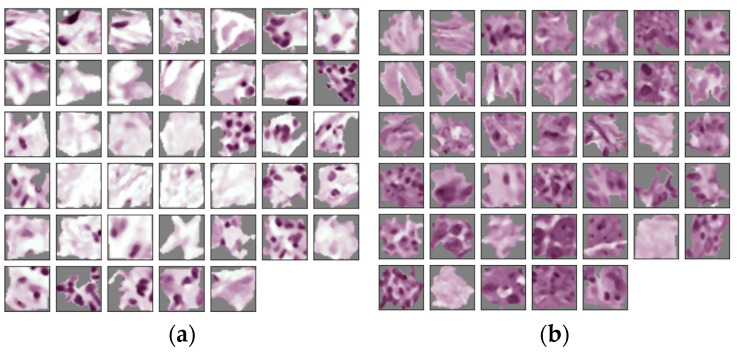
Concept patches from the experiment “feature visualization”. (**a**) Concept for non-melanoma tumors. (**b**) Concept for melanoma tumors. Both concepts contain *n* = 40 patches and seem to share roughly the same cell size, shape, and color. The most obvious criterium to distinguish between the two concepts is the tissue surrounding the depicted cells.

The result of the ACE analysis were two significant concepts ($TCAV_{Q}$: 1.0±0.0). One of them is exclusively for non-melanoma tumors (see Figure 2a). It is characterized by cell nuclei in the tumor-surrounding tissue. The other one is exclusively for melanoma (see Figure 2b). This concept is characterized by cell nuclei in tumorous tissue. For the entire set of concept patches, see Figure A3. The direct comparison of the concepts shows that the CNN not only depends on the shape of cell nuclei. It also relies its decisions on the texture of the surrounding area.

**Figure 3:**
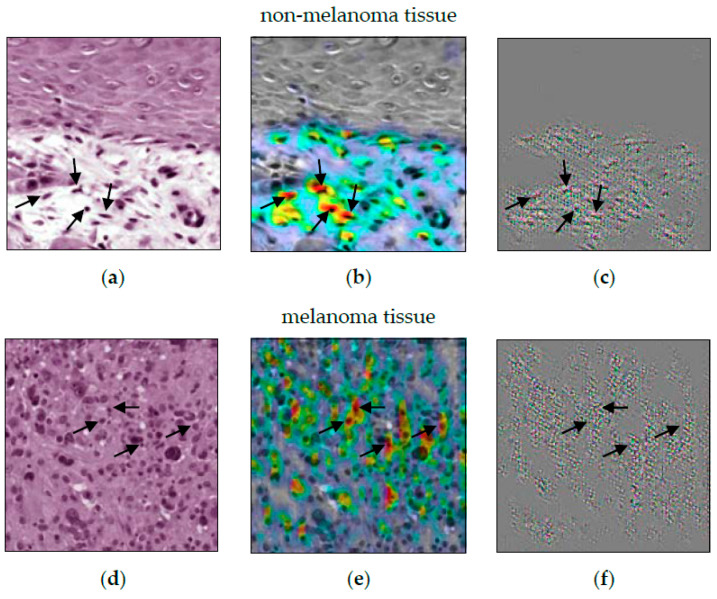
Guided Grad-CAM analysis for the differentiation between non-melanoma tumors (composed of BCC, SCC, and IEC) and melanoma. (**a**,**d**) show an image patch for non-melanoma and melanoma tissue, respectively. (**b**,**e**) show the heatmap analysis. In (**c**,**f**), guided Grad-CAM can be seen. The noise-like structure in (**c**,**f**) leaves room for interpretation as to how they relate to the tissue in (**a**,**d**).

When comparing ACE to Guided Grad-CAM (see Figure 3), it first appears that the CNN decision for non-melanoma tissue is based on the cell nuclei. Especially, Figure 3c shows the nuclei as small black dots. The decision criteria for melanoma tissue are more difficult to understand. In Figure 3e, cell nuclei are still highlighted. Figure 3f just shows a grooved pattern. It is not visually obvious that the CNN distinguishes between cells in melanoma and non-melanoma tumors by the surrounding area. Here, Guided Grad-CAM can be invalid if this is not recognized, and false conclusions are drawn.

Dermatopathological diagnosis based on the concept patches is difficult due to their small size. Since it is out of scope, we do not make a final judgement. Regardless, the results might be problematic from a medical perspective. The model seems to decide based on the area surrounding cell nuclei with similar shape and size as lymphocytic infiltrate. However, both melanoma (superficial spreading and nodular) and non-melanoma skin cancer (IEC and SCC), can show lymphocytic infiltrate [95]. When only looking at the quantitative evaluation, this ambiguity could go unnoticed.

### 3.2. Biases

#### 3.2.1. Class Sampling Ratio Bias

For the class sampling ratio bias, the probability of a tumor tissue sample during training was reduced by 50%, resulting in a distorted tissue distribution. The quantitative performance of the model for class sampling ratio evaluation dropped notably compared to the correct sampling ratio setting. The model achieved a balanced accuracy of 70.8%, an AUC of 0.625, an F1 score of 72.7%, a precision of 80% and a recall of 66.7%. For comparison, the model without a modified sampling ratio achieved a balanced accuracy of 75.0%, an AUC of 0.875, an F1 score of 85.7%, a precision of 75.0% and a perfect recall of 100%. The ROC curves can be seen in Figure A2.

**Figure 4:**
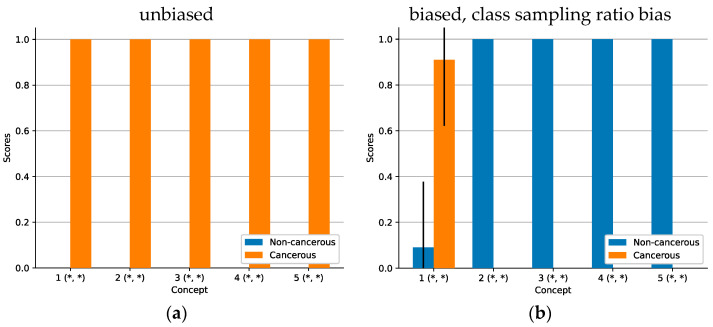
Mean ACE concept scores for different sampling ratios. An asterisk (*) denotes statistical significance of the concept. Standard deviation is depicted as a black line. High $TCAV_{Q}$ scores indicate a high importance of the concept representation extracted by ACE. (**a**) The unbiased model with a correct sampling ratio. ACE explained the model decision using concepts that are related to the cancerous class. (**b**) The biased model with a modified sampling ratio. According to ACE explanations, the model decision is seemingly based on concepts mostly related to the non-cancerous class. A comparison of both figures indicates that the reduction of tumor samples in the training dataset in (**b**) makes the CNN ignore visual indications of tumor tissue. However, those visual cues, as seen in (**a**), are crucial for distinguishing between cancerous and non-cancerous tissue. Due to the bias of the model, the classification performance in terms of the AUC decreases from 0.875 in case of (**a**) to 0.625 in case of (**b**).

**Figure A4:**
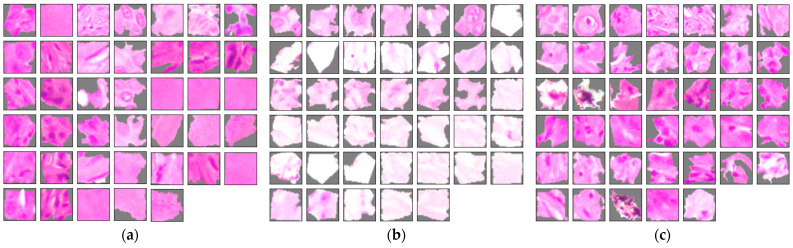

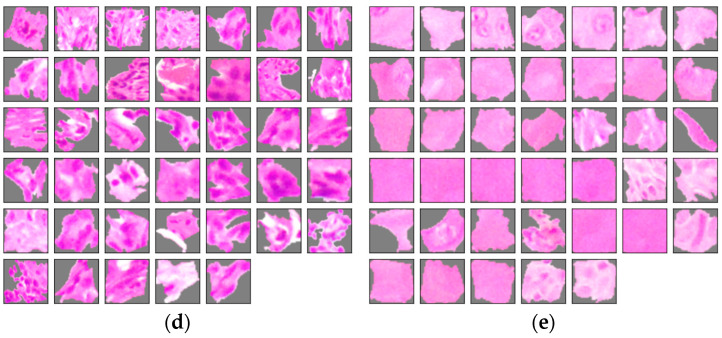
Concept patches from the experiment “class sampling ratio bias”. All concepts belong to the biased model. Thus, they are part of the biased decision logic. (**a**) Concept 1, which is mainly used for the class “cancerous”. (**b**–**e**) show concepts 2–5, which are exclusively used for the prediction of the class “non-cancerous”. Number of patches per concept set to *n* = 40.

ACE analysis revealed five significant concepts (see Figure 4). While the concepts for the model with a correct sampling ratio focus exclusively on cancerous attributes ($TCAV_{Q}$: 1.0±0.0), only one out of five concepts of the other model focusses on cancerous tissue ($TCAV_{Q}$: 0.91±0.29). The four remaining concepts are all classified as non-cancerous ($TCAV_{Q}$: 1.0±0.0). For the entire set of concept patches, see Figure A4.

**Figure 5:**
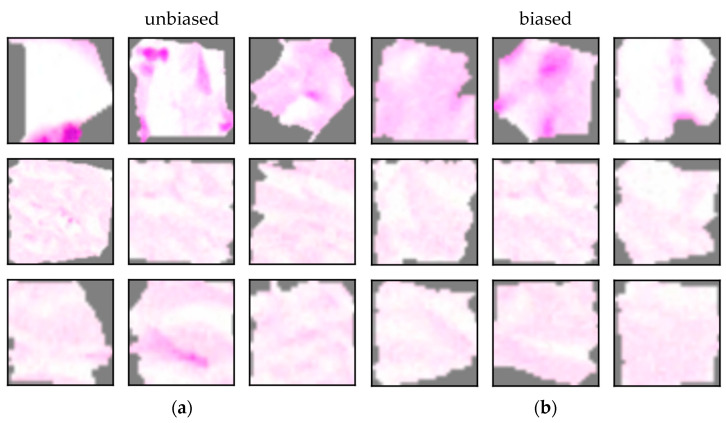
Typical concept patches from different sampling ratio settings. The entire set of concept patches for the model with a modified sampling ratio can be found in Figure A4. (**a**) Concept 1 for the model trained with a correct sampling ratio. (**b**) Concept 2 for the model trained with a biased sampling ratio. Here, the probability of a tumor tissue sample was decreased by 50% during training. Colors and shapes of both concepts appear to be similar. This suggests that both models use the same visual decision criteria, but they draw different conclusions.

The concepts learned by both models look similar (see Figure 5). While the concepts seem to be the same, the model in Figure 4b seems not to have learned to assign them to the correct class. Although, ACE still allows to detect a defect. According to Kempf et al. [95], a histopathologist uses up to seven visual criteria to detect the presence of a SCC. Those criteria are all positively formulated and point towards the presence of a SCC. None of them refers to features for healthy tissue or features as a criterion against SCCs. Consequently, the class “non-cancerous” of the CNN should be defined by the absence of cancerous features only. In other words, the presence of non-cancerous tissue does not guarantee the absence of cancerous tissue. The dominance of non-cancerous features in Figure 4b thus contradicts medical domain knowledge. In contrast, the dominance of cancerous features in Figure 4a is in line with this decision logic.

**Figure 6:**
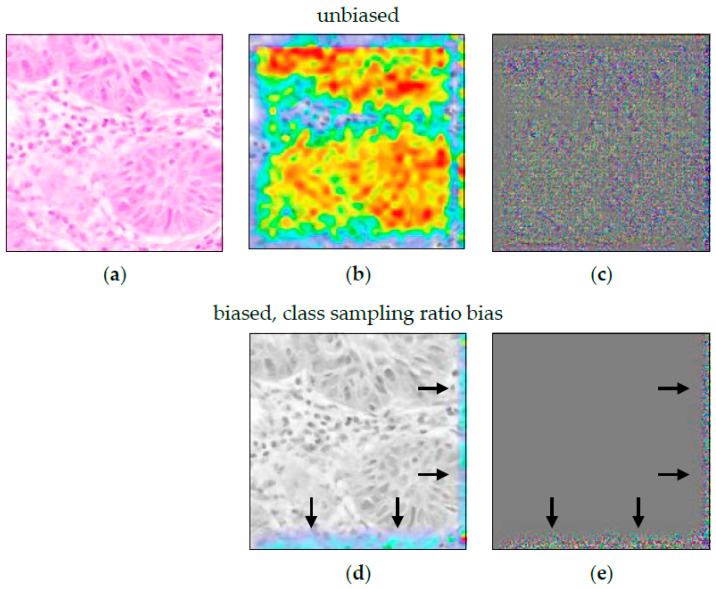
(Guided) Grad-CAM analysis for the cancerous sample in (**a**). The Grad-CAM (**b**) and the Guided Grad-CAM (**c**) analysis of the unbiased model show that the CNN uses features of cancerous tissue. Grad-CAM (**d**) and Guided Grad-CAM (**e**) applied on the biased model are unspecific. Cancerous features, which are relevant for the decision task, are ignored due to the biased training. The analysis confirms the insights in Figure 4.

An exemplary heatmap analysis of a cancerous section is shown in Figure 6. Heatmaps of the false negatives showed that the predictions are based on non-cancerous tissue as well as background. The heatmap of the false positive is spatially unspecific. The highlighted edges in Figure 6d are invalid here. Heatmap analysis alone does not reveal that the model assigns cancerous structures to the wrong class. Thus, while it validly indicates a malfunction of the model, it is invalid in correctly visualizing the nature of the defect in this specific setting.

#### 3.2.2. Measurement Bias

For the measurement bias, images from two different datasets with different image characteristics were combined into one training dataset without any kind of preprocessing such as stain normalization or color augmentation for image adjustment. Regular quantitative performance evaluation of the model for the measurement bias showed that it seemingly achieved a perfect performance. On the test split, it detected malignant tissue with a balanced accuracy of 100%, an AUC of 1.0, an F1 score of 100%, a precision of 100%, and a recall of 100%. The ROC curve can be seen in Figure A2.

**Figure 7:**
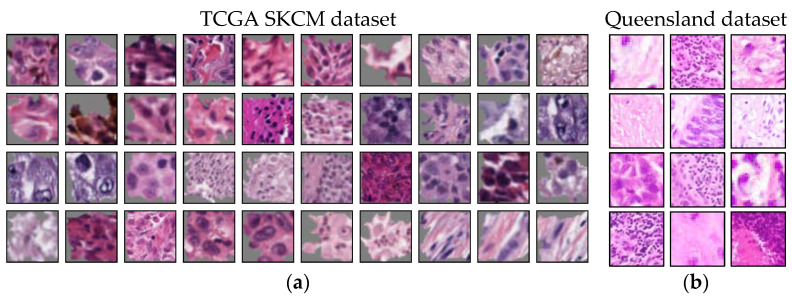
(**a**) ACE concept for measurement bias. The concept patches represent the various stain characteristics present in the SKCM dataset. All patches belong to one scanner/staining setup. This was confirmed by printing a list with the class of all patches. (**b**) Manually extracted image patches from the Queensland dataset. The visual appearance (esp. brightness and color) is different from the patches in (**a**). On average, the images are brighter, and color variation is smaller.

The ACE analysis resulted in one significant concept ($TCAV_{Q}$: 1.0±0.0, see Figure 7a). Further detailed analysis revealed that all concept patches originate from the same dataset. The concept shows independence of cell shape and texture. While it might be hard to visually recognize this in the first place, ACE correctly identified the measurement bias. For direct visual comparison, randomly extracted sample patches are shown in Figure 7b.

**Figure 8:**
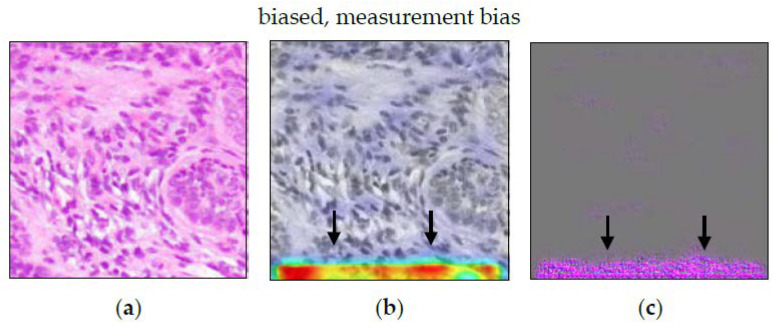
Guided Grad-CAM for model with measurement bias. (**a**) shows an image patch that belongs to the class “non-melanoma tissue”. Grad-CAM analysis in (**b**) highlights the lower edge of the image as the class-specific decision criteria. The Guided Grad-CAM analysis in (**c**) confirms this. Obviously, the H&E staining affects the WSI. However, there are no further indications as to how the findings of (Guided) Grad-CAM relate to the tissue structure and/or texture in (**a**).

The heatmaps in Figure 8 are quite unspecific. Guided Grad-CAM showed a focus on the lower edge of the image. This held true for both classes, malignant and non-malignant tissue. Apart from that, there were no further clues towards the measurement bias. Here, while it validly indicates a malfunction of the model, Guided Grad-CAM is invalid in correctly visualizing the nature of the defect.

#### 3.2.3. Sampling Bias

For the sampling bias experiment, we exemplarily excluded reticular dermis tissue during training. Quantitative performance evaluation of the model with a sampling bias showed a high classification performance. On the test split, it detected cancerous tissue with a balanced accuracy of 87.5%, an AUC of 1.0, an F1 score of 85.7%, a precision of 100%, and a recall of 75.0%. The ROC curves can be seen in Figure A2.

**Figure 9:**
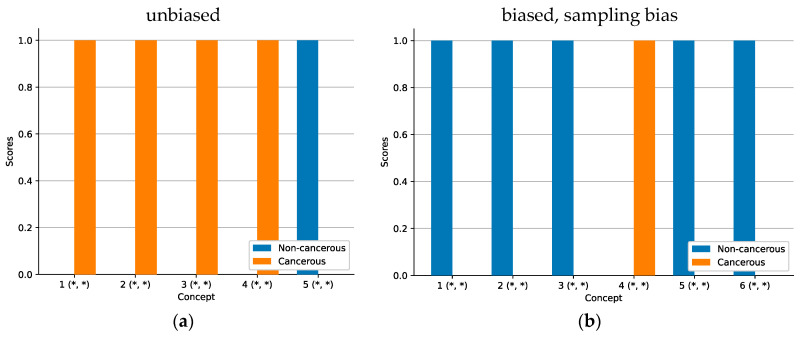
Mean ACE concept scores for the biased model. An asterisk (*) denotes statistical significance of the concept. High $TCAV_{Q}$ scores indicate a high importance of the concept representation extracted by ACE. In (**a**), the unbiased model is mainly characterized by cancerous concepts. The biased model in (**b**) is mainly explained by non-cancer-related concepts. A comparison of both figures indicates that the exclusion of reticular dermis tissue during training in (**b**) makes the CNN ignore visual indications of cancerous tissue. However, those visual cues, as seen in (**a**), are crucial for distinguishing between cancerous and non-cancerous tissue. The classification performance in terms of the AUC is 1.0 for both, (**a**,**b**).

**Figure A5:**
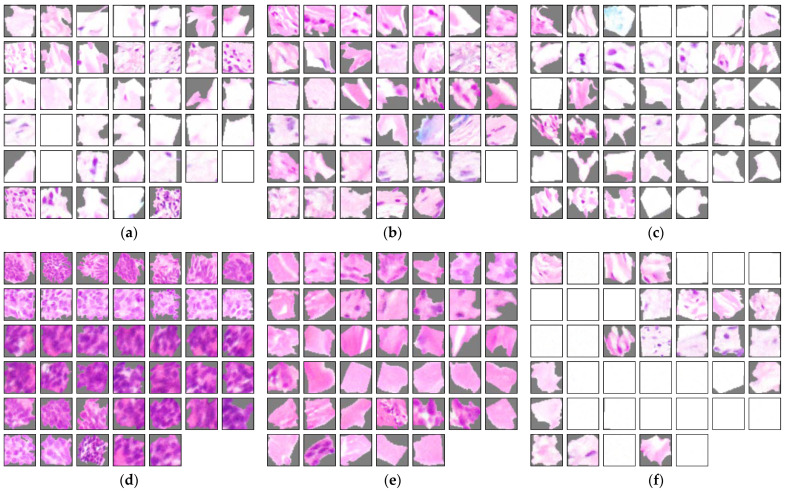
Concept patches from the experiment “sampling bias”. All concepts belong to the biased model. Thus, they are part of the biased decision logic. The concepts 1–3 in (**a**–**c**) as well as the concepts 5 and 6 in (**e**,**f**) are exclusively used for the prediction of non-cancerous tissue. Concept 4 in (**d**) is used to predict the class “cancerous”. Number of patches per concept set to *n* = 40.

The ACE analysis resulted in five significant concepts for non-cancerous tissue ($TCAV_{Q}$: 1.0±0.0) as well as one significant concept for cancerous tissue ($TCAV_{Q}$: 1.0±0.0, see Figure 9). In comparison, ACE analysis of the unbiased model resulted in four significant concepts for cancerous tissue ($TCAV_{Q}$: 1.0±0.0) and one significant concept for non-cancerous tissue ($TCAV_{Q}$: 1.0±0.0). It is conspicuous that there is a shift from cancerous to non-cancerous concepts. The entire set of concept patches for the model with a sampling bias can be found in Figure A5.

**Figure 10:**
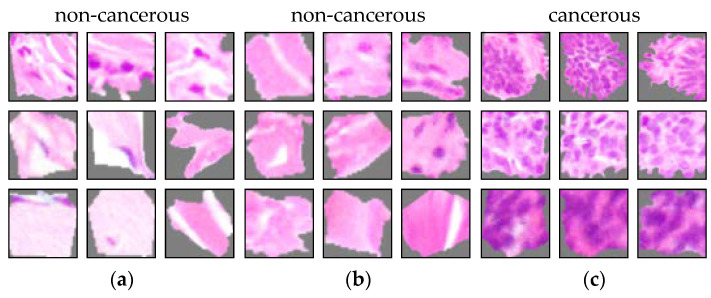
Concepts of a model with a sampling bias. The entire set of concept patches for the model with a sampling bias can be found in Figure A5. Typical concept patches in (**a**,**b**) reflect the increased attention of the model to non-tumorous tissue. However, the presence of non-tumorous tissue does not guarantee the absence of tumorous tissue. Consequently, the correlation with the class “non-cancerous tissue” is probably non-causal. The concept patches in (**c**) represent cancerous cells and are perceptually similar for the biased and the unbiased model.

As described in Section 2, the model was not properly trained on non-cancerous tissue. When comparing the concepts to those of the unbiased model, it is noticeable that there is a shift towards matching concepts (see Figure 10). The concepts in (a) and (b) are used to detect non-cancerous tissue. However, they show collagen tissues from the reticular dermis. Equivalent concepts were not found for the unbiased model. Analogous to the class sampling ratio bias, the predominance of non-cancerous attributes (Figure 9b) contradicts the decision logic of a histopathologist [95]. The presence of collagen tissue does not guarantee the absence of cancerous tissue in an image. Thus, the concepts for collagen tissue are not proper decision criteria for non-cancerous tissue. Remarkably, the bar chart representation in Figure 4b and Figure 9b look similar and do not allow differentiation without additional knowledge. It might thus be invalid to distinguish which type of sampling bias is present in the dataset.

**Figure 11:**
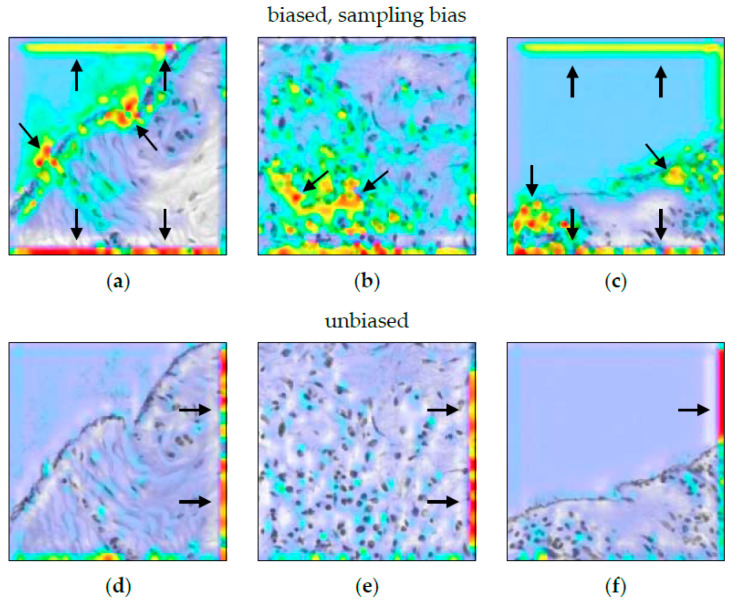
Guided Grad-CAM for model with a sampling bias. (**a**–**c**) show the heatmaps for the biased model. (**d**–**f**) are the equivalent heatmaps for the non-biased model. There are no further indications as to how the highlighted edges in (**a**–**f**) relate to the structure and/or texture of the analyzed tissue section.

When comparing ACE to Guided Grad-CAM (Figure 11), it can be clearly seen that the biased model shows increased attention to non-cancerous tissue, while the non-biased model simply ignores those areas. Thus, it is a valid method in the biased setting. However, the highlighted edges in the unbiased setting are misleading if the histopathologist is not aware of the technical cause.

#### 3.2.4. Class-Correlated Bias

As a class-correlated bias, we replaced a square region in the upper left corner of all cancer image patches with a single color. Quantitative performance evaluation of the model with a class-correlated bias showed a perfect classification performance. The model detected cancerous tissue with a balanced accuracy of 100%, an AUC of 1.0, an F1 score of 100%, a precision of 100%, and a recall of 100%. The ROC curve can be seen in Figure A2.

**Figure 12:**
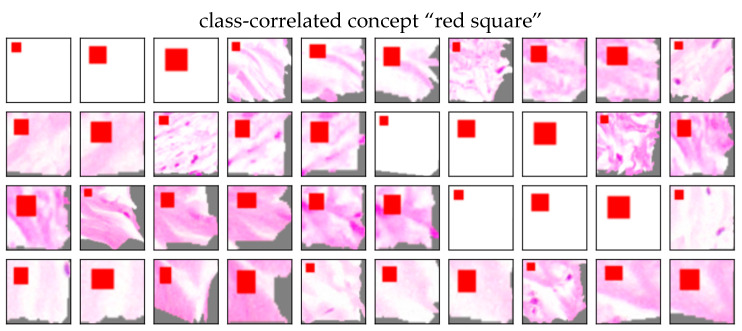
Concept “red square” as an example for a class-correlated biases. All concept patches show the same red square. It is visually recognizable that the square is the decision criteria. In this example, it can be easily concluded that the concept is of non-biological nature. Thus, the correlation to the class “tumor tissue” must be non-causal.

**Figure A6:**
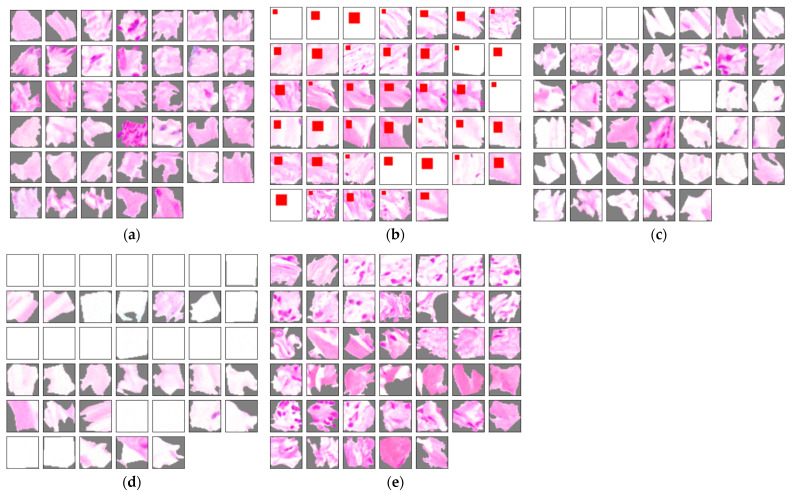
Concept patches from the experiment “class-correlated bias”. All concepts belong to the biased model. Thus, they are part of the biased decision logic. The concepts in (**a**,**c**–**e**) are exclusively used for the prediction of the class “cancerous”. Concept 2 in (**b**), which is rated as “non-cancerous” in half of the cases, clearly shows a red square on all patches. Number of patches per concept set to *n* = 40.

The ACE analysis revealed four significant concepts for cancerous tissue ($TCAV_{Q}$: 1.0±0.0). Another concept got a non-significant mixed $TCAV_{Q}$ score of 0.54±0.48 for non-cancerous tissue ($TCAV_{Q}$: 0.44±0.50 for cancerous tissue, respectively). The specific concept “red square” (Figure 12) clearly shows the correlation between the artificial modification of the image and the class. For the entire set of concept patches, see Figure A6. A histopathologist can easily identify the non-biological nature of the concept “red square”. Even if the bias originates from a biological cause, the histopathologist can visually inspect the patches and decide whether the concept is biologically plausible or not.

**Figure 13:**
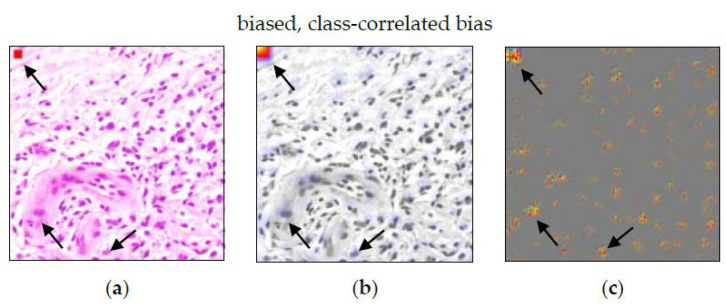
(**a**) Image patch, (**b**) Grad-CAM, and (**c**) Guided Grad-CAM analysis for class-correlated bias. The red square can be seen in the upper left corner of the image. While the heatmap in (**b**) clearly highlights the red square, the visualization in (**c**) is not as specific. Apart from the upper left corner, it also highlights cells spread over the image patch. As can be concluded from (**b**), they are not crucial for the final decision.

When comparing ACE to Guided Grad-CAM, the results are confirmed (see Figure 13). The red square is highlighted by Grad-CAM (b) as well as by Guided Grad-CAM (c). However, the pixel-wise presentation in (c) also highlights other detections, potentially distracting from this specific pattern. When considering both visualizations, it is still valid in visualizing the defect.

### 3.3. Technical Insighs for Histopathology

For concept discovery, we did not normalize the concept patch size as cell size is assumed to be an important feature in histopathology. Thus, scale invariance of the model as described in [55] is not possible. In addition, the feature maps of the convolutional layers are not spatially invariant. Using those feature maps led to meaningless concepts, as ACE grouped patches based on location. This limits the feature vector to the fully connected (FC) layers. We did not notice any drawback of this approach. Fang et al. [61] have also successfully used FC layer features in the context of infectious keratitis.

**Figure 14:**
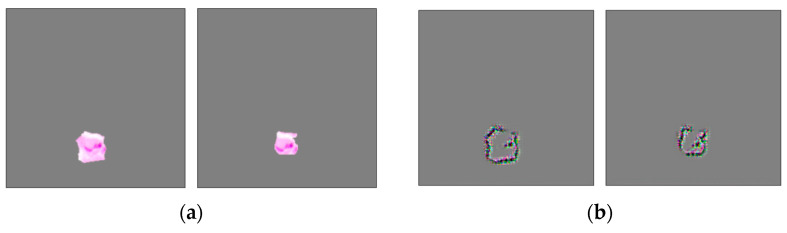
ACE concept patches as extracted by SLIC clustering. (**a**) Patch images of one concept as they are subsequently used for TCAV analysis. (**b**) Guided Grad-CAM analysis applied on those patch images in (**a**). The class-discriminative feature of the concept is the shape of the SLIC clustering, not a biological or medical property of the tissue. This indicates that there is a technical problem regarding the application of ACE. ACE explanations are then related to this problem, not to the actual decision criteria of the model. Consequently, the ACE results are not meaningful.

Ghorbani et al. [55] only stated that they filled the background with a grey scale value of 117.5. This misled our CNNs to classify the SLIC shape as a class-discriminative feature (see Figure 14). The shape seemed to resemble a cancer-specific feature. As a result, the extracted concepts seemed to be random or meaningless. In addition, we noticed that the grey areas of the image can confuse the CNN classification, if these do not occur during training. The results were $TCAV_{Q}$ scores in favor of the wrong class. Those unwanted effects disappeared after using random cutout with a fill value of 127. This way, the CNNs got insensitive to such class-unspecific, grey areas. Furthermore, this is known to reduce overfitting [87]. For a successful application in histopathology, see for example Jin et al. [96]. Thus, one could argue that usage in the context of ACE has a beneficial side effect, as generalization performance should also increase, or vice versa.

As already mentioned by Ghorbani et al. [55], we also experienced duplicate concepts. This originated from the fact that the number of concept clusters must be specified by the user beforehand. A practical solution when noticing duplicate concepts was to decrease the number of concepts. On the other side, we experienced that too few concepts led to mixed, inconsistent concept results, which complicates the interpretation. In this case, increasing the number of concepts helped to increase consistency.

## 4. Discussion

### 4.1. Benefits of ACE over Heatmaps

Heatmap methods have proven valid in histopathology [7]. However, our findings suggest that ACE can offer some technical benefits over heatmaps. Hägele et al. [7] demonstrated that quantitative model evaluation alone is insufficient. They showed that heatmaps can explain the decision process of a CNN on cell-level. It is possible to recognize fine-grained structural details such as nuclei, nuclear membranes, or cytoplasm. However, recognition might also depend on texture. In our experiment, concept-based explanations were more explicit on that. Furthermore, ACE analysis of our model in the feature visualization setting showed that the decision criteria are not automatically in line with established medical decision criteria. While we do not aim to build a medically plausible classifier, the example nevertheless further highlights the importance of model transparency and bias discovery.

Heatmap analysis allows to detect a class sampling ratio bias [7]. However, Grad-CAM of false positives and false negatives did not show the perception of features of the ground truth class. To check whether the CNN completely overlooked the class-specific areas or just weighted them incorrectly, one must generate additional heatmaps for the ground truth class. The detection of a class sampling ratio bias using ACE also appears possible. Here, on the one hand, ACE allowed us to verify which concepts were learned by the CNN. On the other hand, it showed whether those concepts were assigned to the correct class or not. Still, it is challenging to distinguish between this and other bias types. Especially, a sampling bias results in a similar pattern (see Figure 9b).

As shown by Hägele et al. [7], a dataset bias can be detected with heatmap-based methods. In their setup, the bias was spatially located in the image center. Due to its working principle, ACE is at a disadvantage compared to heatmaps for location-dependent tasks [7]. For those cases, it seems advisable to complementary use both algorithms. The same has also been proposed by Graziani et al. [46] and Sousa et al. [39]. Congruent results from both methods might further increase confidence in a model.

In our experiment, the heatmaps indicated a measurement bias (see Figure 8). However, this bias affects the whole image patch. The saliency method failed to visualize this. In addition, when combined with other visual decision criteria, it might be overlooked. For example, the same heatmap pattern (highlighted edges) can be observed for classes characterized by the absence of other features (Figure 11d–f). Tong and Kagal [37] also described the risk of overlooking a bias. In such advanced use-cases, model performance might be erroneously attributed to other criteria. ACE was able to detect a measurement bias as well. Here, the combined evaluation of heatmaps and ACE can give additional indications about the presence of such a non-trivial bias.

Heatmap methods can indicate a sampling bias (see Figure 11). In [7], the model systematically misclassified necrosis tissue. In our experiment, heatmaps similarly indicated a sampling bias (see Figure 11a–c). When the model is not biased, the heatmaps in Figure 11d–f indicates the specific negating decision logic of the class “non-cancerous”. However, this logic affects the whole image patch (respectively, none of it). Similar to as with the measurement bias, the saliency method failed to visualize this. ACE was also able to uncover a sampling bias. Here, concept-based explanation can be useful for better understanding, as the bar chart in Figure 9a is a better representation. The wide absence of non-cancerous concepts equals to the negating decision logic for non-cancerous tissue. We therefore argue that the concept-based representation gives a better insight into such negating decision logics.

Hägele et al. [7] showed that heatmaps reveal a class-correlated bias. However, cells highlighted by Guided Grad-CAM in Figure 13c might be misinterpreted as part of the decision criteria. The only counterindication is the absolute strength of the relevance in (b). ACE also successfully detected a class-correlated bias. In contrast to the heatmaps, the concept in Figure 12 directly presents the relevant shape (red square) to the user. The concept representation is more spatially focused on the actual decision criteria, if other “noise-like” areas are also highlighted on the heatmap. The concept shows that the red square was clearly perceived as an autonomous concept distinct from cells.

### 4.2. Intuitiveness of Concept-Based Explanations

As mentioned before, literature on concept-based explanations postulates a higher intuitiveness compared to saliency maps [46]. While this was also an assumption of our study, we noticed that this advantage did not realize as expected. On the contrary, some of the explanations seemed unintuitive to us. Especially for the class sampling ratio bias (Figure 4) and the sampling bias (Figure 9), perceived intuitiveness of the bar chart visualization seemed very low from a clinical perspective. For the experiments on feature visualization, measurement bias, and class correlated bias, the bar chart visualizations contain little information, so we excluded them from the manuscript.

From a technical standpoint, the visualization fulfills the same requirements as in the studies by Kim et al. [48] or Graziani et al. [46]. Technically, all information needed to detect the bias can be found in the bar chart. However, in the study of Kim et al. [48] on real-world images, concepts such as color (red, yellow, blue, green) or simple texture (zigzagged, striped, dotted) are much simpler and can easily be understood by using common sense. While it would be desirable to acquire such easy explanations for histopathology, we cannot always expect this. In our context, both concepts and classes are much more complex. More pathological domain knowledge is needed to interpret and understand the results. Consequently, especially the bar chart visualizations appeared abstract to us. It seems plausible that this might negatively affect intuitiveness [70]. Some of the semantic and intuitive advantages do not seem to have translated to pathology. Our observation might suggest that the benefits depend on limiting factors such as the application context. A deeper understanding may be necessary for a beneficial use of concept-based XAI techniques beyond ImageNet.

### 4.3. Comparison to Other Bias Detection and Discovery Methods

Part of previous work on bias detection and discovery is not applicable on histopathology, as it has been specifically designed for other use-cases such as faces [16,17]. REVISE [31] is specifically designed for biases associated with objects, gender, and geography, and is thus not transferable to histopathology. DeepInspect [29] is limited to class-correlated biases and thus only covers a small fraction of the requirements in histopathology. In order to apply the methods presented in [18,20], the regions of interest must be known and labeled a-priori. Similar, Zhang et al. [19] require the a-priori definition of attribute relationships. Other approaches require the explicit proposal of bias concepts [27,28,30] and are thus not suitable for bias discovery.

Hägele et al. [7] did a similar study, where they demonstrated the helpfulness of LRP on skin melanoma, breast carcinoma, and lung carcinoma. LRP highly overlapped with expert labels, reflected a predominance of precision or recall, and enabled to detect various types of biases. Our study confirms their findings on pixel-precise heatmaps and adds further evidence for a second, more popular heatmap method, namely Guided Grad-CAM. Based on the systematic DSR approach, we provided evidence for the suitability of two further XAI methods. ACE and Guided Grad-CAM are valid alternatives to LRP methods alone and can be used beneficially in a complementary manner. However, ACE’s intuitive advantages over LRP seem for now to be limited. To completely replace heatmap methods, its intuitive advantages must be leveraged first. We thus think that for now, ACE, Guided Grad-CAM, and LRP should be seen in a complementary rather than a competitive way.

Most other evaluations of XAI methods for bias discovery [36,37,38,39,40] fall into the same category as LRP [7]. They thus suffer from the same disadvantages regarding intuitiveness. Again, further effort on the intuitiveness of ACE is needed to fully replace them.

Previous work already demonstrated the usefulness of CAVs [13,14,23,24] or similar methods [12,15] in bias discovery. However, the reliance on additional models such as GANs for deliberate attribute manipulation introduces additional complexity (e.g., see [15]). Denton et al. [13] thus argued that both approaches might be complementary regarding bias discovery and providing interpretable evidence. Interestingly, the approach by Krishnakumar et al. [26] used a similar approach compared to ACE. Furthermore, they also incorporated Grad-CAM into their method. However, the UDIS algorithm [26] relies on a concept threshold based on accuracy to filter bias concepts. As the authors already mentioned, this is a pitfall as spurious correlations might improve performance and thus remain unnoticed when only relying on accuracy. They noticed that UDIS misses interpretable biases against protected attributes.

Anders et al. [23] recently argued that global XAI methods (such as TCAV) are inappropriate to discover unknown biases. They used this as a justification to bridge global and local XAI (such as LRP, (Guided) Grad-CAM etc.). The previous work in [21,22,24,25] also belongs to this category. Here, we showed that this argument is not valid and global methods can be valid for bias discovery. We thus argue that separate tools for XAI and bias discovery are not always needed, as ACE served both purposes in the evaluated histopathological use-cases.

## 5. Conclusions

Except for location-dependent biases, ACE is valid for bias discovery in computational histopathology. Complementary use of concept- and saliency-based methods can be better than using one method alone. Technical challenges during application can be successfully addressed. For some use-cases, we did not observe the semantic and intuitive advantages of concept-based XAI over heatmap methods, as described in the literature. Our work may help to encourage transparency and reproducibility in computational histopathology in the future.

### 5.1. Limitations

While concept extraction is already automated, some parameters still must be manually set, such as the number of clusters or the concept size (s). This can impact the quality of the results, e.g., in form of duplicate (also see [55]) or mixed concepts. Further, our technical approach described in Section 3.3. limits application to models already trained with random cutout. While linear approximation using directional derivative was sufficient in our study, more complex biases might require more complex representations [53,54,55].

Our research is limited to the criterium validity. While we argue that the reduced complexity in interpretation might improve acceptance, further empirical evidence is needed. While we also argued that congruent results from the simultaneous application of ACE and heatmaps might increase confidence, we do not provide empirical evidence. Although the demonstration of ACE is a first step of evaluation, it does not quantify how well ACE works. For example, one CNN architecture is used based on the assumption that ACE will equivalently work for other architectures.

We assumed that a clinician can distinguishing between relevant and irrelevant decision criteria. When this is not the case, for example for variables with unknown causal links to histopathology, interpreting the results might be challenging. Another limiting factor is the bar chart of ACE, which lacks intuitiveness in the medical context.

### 5.2. Outlook

As we evaluated ACE on several types of skin cancer, future work must demonstrate applicability to other cancer types. In addition, it should be investigated how ACE results change depending on the extent to which a given bias is present [37]. Furthermore, as the results for sampling bias and class sampling ratio bias were quite similar and thus hardly distinguishable, how to correctly differentiate between them based on ACE analysis needs further investigation. The evaluation of ACE in histopathology should be extended by quantitative experiments on how well ACE works, including different CNN architectures.

Future work should focus on algorithmic improvements of concept proposal, so that the process is fully automatic. It should be evaluated whether histopathology benefits from algorithmic improvements over the concept proposal of ACE [56,57,58,59]. Moreover, more complex explanation approaches [51,52,53,54] should be evaluated for histopathology. Furthermore, a completeness measure might make sure that no bias is overlooked [59]. It might serve as a guidance when choosing the number of concepts. In addition, finding a post-hoc solution to the problem of erroneous detection of the concept patch shape would be beneficial to apply ACE to already trained models.

In the future, it should be investigated whether the complementary use with other techniques such as counterfactual augmentation [13,14] might give additional benefits to the histopathologist. Researchers should evaluate and compare the method with other XAI methodologies regarding other quality attributes such as usability. Acceptance and trust of clinicians should be investigated. Finding a more intuitive visual representation of the $TCAV_{Q}$ values to facilitate understanding in the medical context is needed.

Although we used ACE for deductive, theory-driven post-hoc validation of CNNs, it might be interesting to evaluate it for data-driven theory building in medicine [97].

## Figures and Tables

**Table 1:** Experimental design with varying predefined situations. Experimental conditions are varied intentionally to increase confidence in the results. Further description of the datasets SKCM [79,80] (referred to as “Queensland”) can be found in Section 2.4.1.

Experiment	Dataset	Cancer Type(s)	Target Variable
feature visualization	SKCM, Queensland	melanoma, SCC, BCC, IEC	melanoma vs. non-melanoma tissue
class sampling ratio bias	Queensland	SCC	cancerous vs. non-cancerous tissue
measurement bias	SKCM, Queensland	melanoma, SCC, BCC, IEC	melanoma vs. non-melanoma tissue
sampling bias	Queensland	BCC	cancerous vs. non-cancerous tissue
class-correlated bias	Queensland	IEC	cancerous vs. non-cancerous tissue

**Table 2:** CNN architecture based on ResNet [90]. Downsampling is performed by the layer conv3_1 using a stride of 2. Adapted, with permission, from He et al. [90]. Copyright 2016, IEEE.

Layer Name	Output Size	Filters
conv1	128 × 128	7 × 7, 32, stride 2
conv2_x	64 × 64	3 × 3 max pool, stride 2
		$\left[\begin{array}{c} 3\times 3,\;32\\ 3\times 3,\;32 \end{array}\right]\times 2$
conv3_x	32 × 32	$\left[\begin{array}{c} 3\times 3,\;64\\ 3\times 3,\;64 \end{array}\right]\times 3$
	1 × 1	max pool, 16-d fc, softmax

**Table 3:** Summary of the experiments and their outcome. Table adapted from Hägele et al. [7]. Copyright 2020, Springer Nature, CC BY 4.0.

Experiment	Description	Concepts	Validity of ACE	Validity of Guided Grad-CAM
feature visualisation	classification between malignant and non-malignant tissue	Figure 2	features recognizable	features recognizable, possibly misleading
class sampling ratio bias	different class sampling ratios in mini-batches	Figure 5	bias detectable, possibly misleading	bias detectable, possibly misleading
dataset bias	label bias affecting entire dataset, label only depends on patch center	–	bias not detectable	bias detectable
measurement bias	different image characteristics, such as scanner or staining	Figure 7	bias detectable	bias detectable, possibly misleading
sampling bias	exclusion of a tissue type in the training data (here: reticular dermis)	Figure 10	bias detectable, possibly misleading	bias detectable, possibly misleading
class-correlated bias	image feature correlated with one class label	Figure 12	bias detectable	bias detectable

## Data Availability

The results published here are in part based upon data generated by the TCGA Research Network: https://www.cancer.gov/tcga (accessed on 18 May 2022). Data from the University of Queensland is available at: https://espace.library.uq.edu.au/view/UQ:8be4bd0 (accessed on 18 May 2022).
